# Emotional consequences of the COVID-19 pandemic in adolescents: challenges to public health

**DOI:** 10.1590/1980-220X-REEUSP-2021-0424

**Published:** 2022-04-29

**Authors:** Thaianne Cristine Gadagnoto, Lise Maria Carvalho Mendes, Juliana Cristina dos Santos Monteiro, Flávia Azevedo Gomes-Sponholz, Nayara Gonçalves Barbosa

**Affiliations:** 1 Universidade de São Paulo, Escola de Enfermagem de Ribeirão Preto, Curso de Bacharelado em Enfermagem, Ribeirão Preto, SP, Brazil.; 2 Universidade de São Paulo, Escola de Enfermagem de Ribeirão Preto, Programa de Pós-Graduação em Enfermagem em Saúde Pública, Ribeirão Preto, SP, Brazil.; 3 Universidade de São Paulo, Escola de Enfermagem de Ribeirão Preto, Departamento de Enfermagem Materno-Infantil e Saúde Pública, Ribeirão Preto, SP, Brazil.

**Keywords:** Psychological Stress, Mental Health, Nursing, Adolescent Health, COVID-19, Estrés Psicológico, Salud Mental, Enfermería, Salud del Adolescente, COVID-19, Estresse psicológico, Saúde Mental, Enfermagem, Saúde do Adolescente, COVID-19

## Abstract

**Objective:**

To describe adolescents’ everyday activities and emotional consequences related to the COVID-19 pandemic.

**Method:**

Qualitative study grounded in Alfred Schütz’s social phenomenology, involving 22 students at two public schools in a municipality in the state of São Paulo, Brazil. Interviews were conducted, had their audio recorded, and were analyzed according to a thematic categorization.

**Results:**

Five categories emerged from the accounts: i) adolescents’ reaction before the COVID-19 pandemic; ii) emotional consequences; iii) concern about the family; iv) adolescents’ adaptation; and v) fragmentation of social support networks. Feelings such as uncertainty, fear, anguish, anxiety, and lack of motivation, depressive symptoms, and extreme suicidal ideation were reported.

**Conclusion:**

Paying attention to adolescents’ psychosocial needs is essential, especially in face of the possibility of post-traumatic stress as a result of the COVID-19 pandemic. Health professionals’ technical competence combined with sensibility, strengthening of social support networks, and engagement of different community sectors are fundamental for promoting adolescent mental health in the current transition and resignification period following the COVID-19 pandemic.

## RESUMO

**Objetivo:**

Compreender as vivências cotidianas e as repercussões emocionais da pandemia da COVID-19 para os adolescentes.

**Método:**

Estudo qualitativo, fundamentado na fenomenologia social de Alfred Schütz, realizado com 22 estudantes de duas escolas públicas de um município de São Paulo. Realizadas entrevistas audiogravadas que foram analisadas a partir da categoria temática.

**Resultados:**

Emergiram, dos depoimentos, cinco categorias: i) Reações dos adolescentes ante a pandemia da COVID-19; ii) Repercussões emocionais; iii) Preocupação com o grupo familiar; iv) Adaptação dos adolescentes; v) Fragmentação das redes de apoio social. Foi relatada a vivência de sentimentos de incerteza, medo, angústia, ansiedade, falta de motivação, sintomas depressivos e casos extremos de ideação suicida.

**Conclusão:**

A atenção às necessidades psicossociais dos adolescentes é essencial, sobretudo diante da possibilidade da ocorrência de estresse pós-traumático decorrente da pandemia. A competência técnica associada à sensibilidade dos profissionais de saúde, o fortalecimento da rede de apoio social e o envolvimento dos diferentes setores da comunidade são fundamentais para a promoção da saúde mental dos adolescentes, neste período de transição e ressignificação após a pandemia da COVID-19.

## RESUMEN

**Objetivo:**

Comprender vivencias cotidianas y repercusiones emocionales de la pandemia de COVID-19 en adolescentes.

**Método:**

Estudio cualitativo, fundamentado en la fenomenología social de Alfred Schütz, realizado con 22 estudiantes de escuelas públicas de un municipio de São Paulo. Se realizaron entrevistas autograbadas, analizadas acorde su categoría temática.

**Resultados:**

De los testimonios, surgieron cinco categorías: i) Reacciones de adolescentes frente a la pandemia de COVID-19; ii) Repercusiones emocionales; iii) Preocupación por el grupo familiar; iv) Adaptación de los adolescentes; v) Fragmentación de redes de apoyo social. Se informó presencia de sentimientos de incertidumbre, miedo, angustia, ansiedad, desmotivación, síntomas depresivos y casos extremos de ideas suicidas.

**Conclusión:**

Atender las necesidades psicosociales en adolescentes es esencial, particularmente ante la posibilidad de estrés postraumático derivado de la pandemia. La competencia técnica, asociada a la sensibilidad de los profesionales de salud, el fortalecimiento de la red de apoyo social y la participación de los diferentes sectores de la comunidad son fundamentales para promover la salud mental adolescente en este período de transición y resignificación posterior a la pandemia de COVID-19.

## INTRODUCTION

Public health crisis scenarios such as the new coronavirus (SARS-CoV-2) pandemic, which emerged in China in December 2019, bring along a variety of risks to the health of the population around the world that are not limited to the dissemination of the disease and include increase in prevalence or worsening of mental disorders^(1)^. Because children and ado-lescents are inserted in a phase of the life cycle that is marked by critical development, they are a group that is more vulnerable to the psychosocial effects of the COVID-19 pandemic^(2)^.

Adolescence is a developmental period characterized by multiple biopsychosocial changes involved in the complexity of the transition to adulthood. It is a step marked by high sensitivity to social stimuli and a higher need for interaction with peers^(3)^.

The drastic modifications in lifestyle that resulted from social distancing measures made necessary by the COVID-19 pandemic, and which were implemented abruptly and included temporary closure of schools, services, and recreational activities, originated a dismal social scenario with the potential of affecting the development and well-being of an entire generation of children and adolescents around the world^(4)^.

Exposure to acute and chronic stress, concerns about relatives and economic conditions, unexpected mourning, and increase in time spent on the internet and social media^(5)^, especially under lack of supervision, which implies higher chances of exposure to sexually inappropriate content and vulnerability to fraud and abuse in digital media^(2)^, stand out among the several consequences of the COVID-19 pandemic for adolescents. Some authors considered the possibility of negative impacts on academic performance and development of or increase in restlessness and aggressiveness^(2)^. In addition, social distancing can mobilize internal changes or stir up issues that underlie the pandemic, which triggers the development of psychiatric conditions^(6)^.

Although home should be a place of safety for children and adolescents, the occurrence of physical, psychological, and sexual violence is a relevant fact that has to be stressed, especially because of the increase in the number of cases of violence against women, children, and adolescents during the pandemic caused by SARS-CoV-2^(4)^, worldwide and in Brazil^(7)^.

The consequences of this “parallel pandemic” affect more intensely children and adolescents who live in poverty and are subject to social vulnerability, primarily marginalized social groups and minorities^(4)^. Additionally, lack of recognition and interventions during the social isolation and distancing period exposed the urgent need for new strategies to approach mental and psychological health^(1)^, mainly in children and adolescents.

The objective of the present study was to describe adoles-cents' everyday activities and emotional consequences related to the COVID-19 pandemic. The results can contribute to a better understanding of this population's emotions and responses before the challenges posed by the COVID-19 pandemic daily life, given that there are several gaps in the knowledge in this area, and inform reflection on the subject and formulation of individual and collective care plans that take into account the experience context, intersubjectivity, and adolescents' psychosocial needs. These plans should emphasize the strengthening of

social support networks, consider the possible emotional consequences in the post-pandemic period, and reckon with the need for adjustment and imminent development of post-traumatic stress disorders and long-term consequences in this generation of adolescents.

## METHODS

This was a qualitative study grounded in Alfred Schütz's social phenomenology. This theory seeks to elucidate the action of agents in the social world according to the intersubjective relationships molded during their everyday experiences^(8,9)^. The changes in daily life and the intensity of the routine of adolescents during the COVID-19 pandemic motivated the use of this theoretical framework. It is assumed that social interaction^(3)^ is an essential component for maintaining adolescent health (intersubjectivity and intentionality), and curtailing it by implementing restriction measures aiming to contain the dissemination of the virus may have caused emotional effects.

This theoretical framework is not limited to examining individual conduct. Instead, it looks into the constitution of a typical characteristic of a certain social group that is experiencing a situation. The “reasons for” refer to meeting objectives and expectations and undertaking projects, whereas the “reasons why” pertain to the background or previous experiences in the biopsychosocial sphere^(9)^.

Despite their individual characteristics, adolescents engage in conducts that are typical of the social group to which they belong. To understand their acts, it is necessary to know the “reasons why” they adopt these conducts so it is possible to typify the group's social actions. However, adolescents also have private interests. Therefore, their choice of following or not a certain typification is closely related to their private relevance hierarchy. This means that adolescents, guided by their “reasons why” and “reasons for”, can adopt a stance that differs from that associated with the typification of their social group^(10)^.

### Local

The present study was carried out in a small municipality located in the interior of the state of São Paulo, Brazil.

### Population and Selection Criteria

Adolescents of both genders who had access to the internet and were enrolled in two public schools were included. The invitations to participate were sent to all the students in the chosen classes by means of the schools' WhatsApp groups and had information about the study. The number of participants was determined by applying the data saturation criterion, which manifests as redundancy in the accounts based on the relevance of the contents pertinent to the study object^(11)^. This procedure resulted in a sample of 22 individuals. Six adolescents declined the invitation during participant recruitment.

### Data Collection

Data were gathered between October and December 2020 by conducting semi-structured interviews in online meetings that were streamed and recorded using Google Meet. The data collection instrument was based on a semi-structured script with two parts: the first was designed to provide a sociodemographic characterization of the participants, including information such as age, gender, level of education, and housing conditions. The second was a set of open-ended questions related to the emotional events experienced by the adolescents during social isolation and had the following guiding questions: Regarding your emotions, how was your social isolation period resulting from the COVID-19 pandemic? What were the main difficulties you faced staying home during this period? What did it feel like to spend all this time at home with your family? Did you manage to carry out activities that made you feel good physically or mentally?

The interviews were conducted by a nursing undergraduate under the supervision of two professionals with a PhD in nursing and a PhD student, all of them experienced in qualitative research. The interviewer had already interacted with the analyzed population because she gave lectures about mental health in pandemic times. The interviews were scheduled for days and times that were convenient to the adolescents and occurred as individual meetings between a participant and one of the study authors. The conversations were recorded using a resource that is part of the Google Meet platform after verbal authorization of the participant, which reinforced the consent documented in free and informed agreement forms and free and informed consent forms signed by the adolescents' parents or legal guardians.

A pilot study was carried out with two participants, after which no changes were made in the recruitment process or data collection instrument. Each participant was interviewed only once. After data collection, the interviews were transcribed. The contents of the accounts were organized by using the full transcription of the audios as the source material. The participants' gestures and verbal and nonverbal expressions were kept in the transcriptions. Data confidentiality and participant anonymity were ensured by identifying the interviews with the label A (for “adolescent”) followed by a number assigned to each participant (A1-A22).

### Data Analysis and Treatment

Data codification was carried out manually by two rese-archers, and a third one was consulted when they disagreed. Therefore, the data were categorized and analyzed by following the steps indicated in a theoretical study grounded in Alfred Schütz's social phenomenology^(8)^: the accounts had their audio recorded; the material was fully transcribed; and the interviews were read and reread so the researchers could grasp the adoles-cents' experiences during the COVID-19 pandemic and their consequences. The relevant aspects found in the accounts were grouped to make up the categories, and these were analyzed in an attempt to shed light on the subjective experiences of the adolescents in the “world of everyday life” in the pandemic con-text and their emotional consequences. Five thematic categories were identified: i) adolescents' reaction before the COVID-19 pandemic; ii) emotional consequences; iii) concern about the family group; iv) adolescents' adaptation; and v) fragmentation of social support networks. These categories were submitted to comprehensive analysis and discussion of the results by using the concepts of social phenomenology and the literature in the area as a starting point.

### Ethical Aspects

The proposal was approved by the Ribeirão Preto College of Nursing Research Ethics Committee as per report no. 4.354.107 of 2020. All the participants and their legal guardians were informed about the objectives of the study and signed free and informed consent forms, and all the adolescents signed free and informed agreement forms before data collection. In addition, the Consolidated Criteria for Reporting Qualitative Research Guidelines^(12)^ were applied to observe methodological rigor, and the guidelines proposed in Brazilian National Health Council Resolution 466/2012, on human research, were strictly followed.

## RESULTS

### Sociodemographic Characteristics

The sample was 22 adolescents from 14 to 18 years old, with an average age of 15.68 (±1.04) years, who were enrolled from the ninth grade of elementary school to the third grade of high school at two public schools in a small municipality. Most were female (n = 17; 77.25%). Ten participants declared that they had brown skin (45.45%), seven were white (31.81%), four were black (18.20%), and one declared that they had yellow skin (4.54%). Eleven were Protestants (50.00%), nine were Catholics (40.90%), and two had no religion (9.10%). Most lived in a house owned by their family (n = 16, 72.72%), and the average number of people living in the house was four.

Five thematic categories were identified: i) adolescents' reaction before the COVID-19 pandemic; ii) emotional con-sequences; iii) concern about the family group; iv) adolescents' adaptation; and v) fragmentation of social support networks.

### Adolescents' Reaction Before the Covid-19 Pandemic

It was found that, at the beginning of the pandemic, the adolescents felt less concerned about the events and took them less seriously. The situation was experienced as comfortable, mainly because their classes were canceled. This was initially interpreted as a respite, a “vacation period”, especially when the activity overload reported by some participants was taken into account. Over the period of social isolation and distancing, the adolescents' perception changed, and they began experiencing fear, loneliness, frustration, anxiety, boredom, and anguish before the events that they could neither control nor predict. They also had to live with the drastic changes imposed to their everyday lives.

*In the beginning, I thought it was cool. I said: Oh, I am going to stay home, right? But then a month went by, two months, and I started missing people, you know? I began missing contact with my friends, we did not meet anymore. Then that closing thing began and I said: My God, what is going on? We spent time on social media and saw bad things only, you know? People dying, so it was scary, you know?* (A17)

*Before the pandemic, I had responsibilities other than just school, so I was getting overloaded. Then, when the pandemic began, everything stopped and I was more at loose ends, but now things are getting back to normal. For instance, my performance in the online classes was not as good as I expected, so I think the situation is affecting me more now than in the beginning of the pandemic.* (A3)

*Oh, it was nice, but not so much, because I was a little stressed out about school and stuff and I wanted to stay home for some time. In the beginning, it was kinda good, but now it is lousy, horrible! I cannot stand staying home anymore.* (A12)

### Emotional Consequences

During the first stages of experiencing the COVID-19 pandemic, there was a feeling of normality and even calmness, and the severity of the facts was less perceived and raised less concern. As the pandemic unfolded, worries and questions regarding the future emerged, as well as emotional discomfort and consequences, which brought along the onset or worsening of symptoms of anxiety, sadness, and lack of interest in usual activities.

*In the beginning, it was actually easy because I did not know that it was going to last so long. I thought it was going to last a month, two months maximum, then I realized it was taking longer than the time I had in my mind and things started getting worse (...) anxiety crisis, I began crying more than usual and getting terrified (...). I do not have many friends, what I have is mostly school colleagues. I did not talk much, and during this period I talked even less, then it got worse. I really did not know what to do, you know? No (...). Yeah, I gradually lost interest in the things I liked to do the most, simply because there was no one to do them with.* (A16)

Implementing safety measures, including social distancing, was seen by the adolescents as an imposition or a mandatory act that changed their daily lives and hurt their autonomy, freedom, and power to make decisions. The confinement at home caused anxiety, frustration, restlessness, and anguish, as well as the need to spend more time around the family.

*I was distressed because we could not go out. They made us stay at home no matter what. If things were normal, we could choose between going out and staying at home, so I was upset because I was not allowed to go out and do normal stuff.* (A1)

*I got really distressed, because it is really difficult, like you had a life that was spending nearly the entire day out, and overnight I did not go out even for grocery shopping. It was really hard.* (A8)

Previous psychosocial problems of the adolescents got worse, which contributed to greater mental distress.

*I was upset about not being allowed to go out. Horrible, isn't it? Because staying confined at home and not seeing anyone. like, jeez! I already had issues related to this before the pandemic, so when it started and I had to be quarantined it was even worse. To me, it was awful.* (A5)

The intensity of the emotions was mentioned by the adolescents, especially before the scenario of uncertainty and unpredictability regarding the future and their own perspectives.

*Loneliness, boredom, lack of social contact, the monotonous everyday life, doing the same thing every day, and lack of perspective on the future.* (A14)

There was a discontinuation of the adolescents' plans and wishes for 2020, especially in face of the expectations of a new year and the way adolescents typically experience this period, with relevant changes in social relationships and plans for the future. The sensation that the year that went by was lost, the feeling of frustration, and the difficulty establishing new goals and plans stood out.

*I thought 2020 was going to be pretty different, I was expecting it to be a very good year, you know? Because it was the beginning of a new life, like a new decade ahead (...) So I was hopeful there would be lots of good things, at school and stuff. It was because, like, I have never had many friends so I said to myself: this year, I want to make more friends, when the year is almost over I want to be able to say: Wow, next year I will still be in contact with lots of people. But then the pandemic began and I ended up losing contact with many people I talked to and (...) with my friends. I ended up getting lonelier and a lot of things I did not want to happen ended up happening, you know? Like, it is as if life has actually stopped. And the feeling that I lost the entire year. It seems that I think about that every day, that I lost the entire year, and I do not know what to do. And it is kinda hard to make a decision now because I do not know what I am going to do, like, I cannot explain it very well, but things got really worse.* (A14)

Considering the effects of the pandemic on adolescents with previous psychosocial problems and the worsening of mental health conditions is fundamental. These issues increase the chances that adolescents react to this fragility context by experiencing the onset of a mental crisis, including the manifestation of suicidal ideation.

*I wasn't standing it three months after it started only—I just felt like killing myself! I have (...) Now I can talk about this: I have six farewell little boxes. It has been horrible one way or another since the pandemic broke out. Sometimes I do not even feel like getting out of bed.* (A5)

*I could not sleep. I just cried.* (A17)

### Concern About the Family Group

The participants expressed their concern about their relati-ves before the impact of the pandemic's lethality, which shows that the adolescents had to deal with the fear of death, losses, and mourning, leading to the intensification of their stress and anxiety.

*It was hard getting used to it, I think. It was a shock. In the beginning, I was afraid to lose someone, I think that was the most despairing thing, (...) the fear of losing a family member was the most nerve-racking thing.* (A1)

*It was worrisome, because our country was in a situation in which everyone was dying and a virus was spreading too fast. So I ended up getting really worried about my family, some of my relatives are in the risk group and all. It was also a moment of reflection for me, because we have to enjoy being around people while they are still here, right? After they pass away, it is too late.* (A21)

Some of the adolescents' relatives were health professionals. The participants expressed their concern about the exposure to which these people were submitted and the risk of getting the disease in health services, especially when the family members were professionals in the frontline. Positive cases among health professionals impacted the adolescents' experiences.

*I was afraid because my mom works in a hospital, so the fact that she is there, ready to work, is already a huge risk. She was contaminated with the virus. And I was afraid because she saw many healthy people dying, being contaminated. It was hard for me. I thought something was going to happen to her because she got really sick. It all turned out well, but I was really afraid.* (A8)

### Adolescents' Adaptation

Before the challenges posed by the pandemic, the adoles-cents sought ways to adjust their everyday lives to feel better. However, lack of acceptance and support by other people and the absence of direction in coping strategies hindered the for-mulation, planning, and implementation of effective actions to improve adolescent well-being, which caused the situation of discomfort and distress to persist during the pandemic.

*Actually, I tried to do something that made me feel good, but when you feel really anxious, even if you try, it seems that your efforts are not enough.* (A5)

*Staying at home and not having what to do is very distressing, you feel bored. As much as you can go on the internet, use your cell phone, you cannot do that a hundred percent of the time.* (A19)

Adaptation strategies came up individually and originated in the initiatives of the adolescents themselves, in a field of experimentation, with hits and misses. The main challenge was changing habits, and the most frequent resource to deal with it was spending more time in activities to get some distraction. Going online and using social media for longer periods stood out, which contributed to the persistence of negative feelings.

*I have never really enjoyed being confined in a place for too long. It makes me feel very lonely, although I spent a lot of time on social media. I started doing this when the pandemic broke out so I could feel more connected to the world, but I still felt lonely somehow.* (A5)

*I think it was hard because I had no one to talk to. Before the pandemic, I was always surrounded by many people, at school, whatever I would go on social media, like Facebook or Instagram, and all I would see was bad things, only people dying, no vaccines at all, the government did nothing, things got more and more unstable. I would say: Jeez, when is this all going to be over, get back to normal? So I think the hardest part was staying at home and not having anyone all the time.* (A8)

### Fragmentation Of Social Support Networks

Although the pandemic allowed adolescents to spend more time with their relatives, there was a feeling of isolation in the home environment and fragmentation of bonds and social

support networks. The media and the internet were frequently used resources during this period of crisis and hardship. However, they proved to have a momentary impact and low problemsolving capacity.

*As I did not see people anymore, I talked very little. People at home are kinda “boors”, you know? They are not really fond of talking. It was, gee, (...) very difficult to (...) figure out what was going on, so much so that I started visiting pages providing help on Facebook, that kinda stuff, so I could have someone to talk to, you know?* (A16) *Usually, when I am having a crisis, I try to call someone, I try to talk to someone. A good part of my friends come to my aid, help me. Sometimes it is not possible, right? I try to help myself, but it is pretty complicated, huh? A pretty complicated situation.* (A5)

## DISCUSSION

The present study exposed the experiences of adolescents in face of a singular and challenging scenario: an emerging and unprecedented global pandemic. The participants showed changes in their perceptions over the pandemic period, with less apprehension when the events began unfolding. This scenario was modified by the imminence of fear, loneliness, frustration, anxiety, boredom, and anguish. The intense concern about their relatives' health originated emotional overload, as well as the demand of facing mourning and fear of death.

The COVID-19 pandemic caused an abrupt interruption in the adolescents' plans and goals and resulted in lack of interest in usual activities and worsening of mental health conditions, preexisting psychosocial problems, and even evidence of suici-dal ideation. The worries about the future exacerbated mental distress, a consequence of the unpredictability and uncertainty ahead. The observed adaptation strategy that stood out was the often frustrated attempt of establishing routine and self-care measures, with an intensification in the use of the internet and social media. Additionally, the participants had to deal with an insufficient family support network and problems in acceptance and orientation of effective strategies to promote mental health and maintenance of well-being.

Understanding the experiences, emotional consequences, and nuances of the subjectivity present in the everyday life of adolescents amid the stress arising from the pandemic is fun-damental for planning care and promoting mental health in this population.

The advent of the COVID-19 pandemic broke down and intensely transformed the “world of everyday life” as it was pre-viously structured. School closures, lack of contact with peers, and more time spent at home with the family group caused important and impactful changes in the adolescents' “world of everyday life”, who were in full interactive engagement with a great number of people, in complex social relationship networks.

The interpretation and awareness of the established reality in this *new* “world of everyday life” influence how agents place themselves in it, that is, their “natural attitude”^(9)^. Because this is ruled by a “pragmatic reason”, the “world of everyday life” can be modified by our actions and modify them in a process of continuous transformations based on the ability to naturally intervene in this world, affecting it and being affected by it^(9)^.

Therefore, it was found that, in the view of adolescents, as a group typification, the pandemic was initially associated with lack of concern and a reduced perception regarding the seriousness of the problem. However, over the course of the events and with the impact on the daily life caused by the confinement at home, anguish, fear, uncertainty, lack of safety, anxiety, and sadness became recurrent.

Clinical observations showed that, in the beginning of the pandemic and implementation of lockdown-related measures, there was a collective sense of resilience, with changes such as dealing with online learning, taking up new hobbies, and develo-ping other interests in the domestic environment. In the second phase, there was a more realistic perception regarding questions related to finances, relationships, and education, among other aspects. Subsequently, greater anxiety became patent, with a feeling of tiredness and exhaustion before the situation and increasing frustration and impatience with the multiple effects of the pandemic on people's lives. However, the fear of the virus was a constant in all these phases^(13)^. The experienced abrupt changes had the potential to cause frustration, annoyance, emotional disconnection, nostalgia, and boredom resulting from social distancing^(14)^.

A study showed that, when adolescents are faced with frustrated expectations, such as those related to the pandemic duration, which was longer than initially expected, they were vulnerable to anxiety and depressive symptoms^(15)^. In these cases, the impact of the pandemic on adolescent mental health was more evident in the long run. The main explanation is that, in adolescence, emancipation from the parents occurs progressively, unlike what happens in childhood. Therefore, in the teen years, interaction with peers gets more relevant, with the development of more complex social relations^(3)^. A closer family relationship may also favor situations that stress interpersonal connections^(6)^.

Adolescents are in a “determined biographical situation”. Their experiences cover their stock of experiences (“reasons why”), which can be better understood by knowing their life story, what made a specific agent carry out certain actions or adopt certain conducts^(9)^. Factors that preceded the pandemic include the adolescents' family context complexity, the diffi-culty having a relationship and developing friendship bonds with peers, and preexisting conflicts and mental disorders, among other past experiences that directly influenced the ado-lescents' spontaneous interpretation during the pandemic daily life. A study showed that adolescents who live in highly func-tional families and have positive interpersonal relationships with their parents or legal guardians were less likely to suffer the effects of social distancing measures in comparison with adolescents who did not have positive family relationships or lived alone^(3)^.

The pandemic situation can cause irritability and fear before the possibility of contamination of family members^(6)^ and close people, a finding corroborated by the present study, even among adolescents whose parents were health professionals who were frontliners in the fight against COVID-19. This additional stress made the adolescents feel more insecure and increased the levels of anxiety they experienced^(16)^. Pessimistic perspectives on the pandemic, including fear of being infected or having close people infected, can result in changes in adolescents' conduct and affect their mental health^(17)^.

Adolescence is a period marked by greater vulnerability to mental health problems. Approximately 75% of the adults with psychic disorders showed their first symptoms before they were 24 years old. It was reported that problems in relationships with peers, rejection, bullying, and loneliness are risk factors for developing depression during adolescence^(2)^, suicidal ideation, self-injury, and eating disorders^(18)^. The recurrent verbalization of the feeling of loneliness in the accounts of the participants of the present study reinforced this picture.

Restrictions in mobility and contact with friends can cause stress and result in manifestations of aggressiveness and disobe-dience, as well as intensify the attempts to interact with friends by using digital media^(6)^. Historically, the COVID-19 pandemic is the first experienced in the online era. Digitally mediated interactions cause intense changes in the traditional conceptions of socialization^(2)^. While the internet makes global realization possible, it also raises expectations and paranoia before the large number of sick or dead people that is divulged daily. It is known that the massive consumption of content about the pandemic situation can trigger anxiety and panic and lead to depression^(19)^. In addition, adolescents can have difficulty dealing with or critically and accurately analyzing information shared on social media, which may be untruthful or inexact^(20)^. In the present study, it was found that overexposure to pandemic-related contents caused or aggravated discomfort and negative feelings in adolescents.

Excessive use of the internet can originate addiction^(21)^, that is, a disorder that causes dependence. It can manifest as one out of the five forms that are internationally cataloged: cybersex; relational (related to social media); net gaming addiction, which includes gambling, videogames, shopping, and obsessive e-commerce; search for information; and game addiction^(19)^.

The context of this intensification in digital sociability and use of the internet, especially in a context of social isolation, has the potential of increasing the vulnerability of children and adolescents to self-inflicted violence^(19)^. Therefore, the COVID-19 pandemic scenario requires an extended approach to adolescents that dialogues with the different social phe-nomena in which this population is inserted and takes into account the determining and conditioning factors involved in the health-disease process.

From Alfred Schütz's social phenomenology perspective, nursing care is considered a social action based on the establish-ment of intersubjective relationships that must be recognized as important by nurses in the different settings and contexts in which they carry out their activities^(20)^. Nursing care must take into account the set of knowledge and experiences acquired throughout life and the biographical situation in which agents are when care is provided^(20)^.

By considering the Brazilian history of care actions in the field of mental health oriented toward children and adolescents, which are greatly marked by omission, exclusion, and emphasis on institutionalization, as well as the number of adolescents, the following question arises: Are we ready to deliver mental health care to a generation of adolescents during and after the COVID-19 pandemic? Numerous challenges for public health remain. Additionally, this area is one among many in nursing that make up comprehensive adolescent care.

Amid the major changes in care practices imposed during the COVID-19 pandemic, training of primary healthcare professionals based on an inviting, thoughtful, and observant perspective will be able to evaluate and identify cases of post- traumatic stress, depression, and other mental disorders resulting from pandemic crises^(23)^.

In this context, it becomes indispensable to develop skills to manage first care in the psychosocial sphere, which encom-passes active, attentive, and non-judgmental listening, in order to preserve adolescents' basic needs and reduce their vulnerability^(23)^. In addition, professionals must manage the acute stress that adolescents and their families may show during or after the pandemic, seeking to implement interventions suitable for controlling the levels of stress and anxiety in this population, such as meditation and yoga, among others^(23)^.

It is recommended that primary healthcare teams develop educational actions and promote health by using online strate-gies, given the satisfactory adherence of adolescents to comple-mentary care modalities. The goals would be clarifying health measures to prevent and control the infection and encouraging social responsibility, which presupposes self-care and caring for others^(6)^. Phone and video calls have been used as a way to develop coping strategies with adolescents. However, the limitations resulting from this approach include loss of personal connection and identification of nonverbal clues^(13)^.

It is important to stress that the role of health professionals who carry out their activities in primary health care with ado-lescents in a pandemic and post-pandemic scenario involves an extended perspective and family care, since the emotional consequences experienced by this group may be related to difficulties in the interaction with relatives. In this axiom, it is pertinent that professionals can mediate the conflict friendly and guide parents or guardians to practice active listening of the adolescents' demands, understand their current situations, identify the behavioral changes that are inherent in this phase, and indicate the search for dialogues with other professionals and members of the support network, including parent groups and church, among others^(23)^.

The development of group activities to promote the mental health of adolescents and their families, whether in online environments or face-to-face meetings by observing nonpharma- cological preventive measures against SARS-CoV-2, in which they can express themselves freely, allows the creation of a space of listening and embracement, with the participation of primary healthcare professionals. The group activities can allow the gathering of subjectivities, with the recognition of common aspects and sharing of adolescents' experiences in the “world of everyday life” in the pandemic context and their expectations about the future (“reasons for”). These events can become a therapeutic space that favors bonding, reflection, and opportunities for adolescents, their relatives, and health professionals to enrich their lives.

The present study has a major limitation. There was no differentiation of the participants regarding preexisting health conditions, including mental health disorders. However, the used method allowed the adolescents to outline their everyday conflicts and reflect on their care needs and their implications in the relevance of promoting adolescent mental health.

The theoretical framework in which the present study was grounded, social phenomenology, brought to the surface the importance of the intersubjectivity present in the experiences of the adolescents in the “world of everyday life” transformed by the COVID-19 pandemic. It also emphasized the “reasons why” and the “reasons for” related to the consequences of the pandemic for the adolescent public, a group that was left on the sidelines of the discussions about the main guidelines adopted in the pandemic context. Last, it illustrated the importance of implementing measures oriented toward protecting adolescent mental health by using an intersectoral approach. By dissemi-nating the contents of the present study and adopting strategies that meet the needs of the target public, it is possible to present several psychosocial and somatic outcomes in the short, medium, and long run.

## CONCLUSION

The COVID-19 pandemic led to deep emotional con-sequences in adolescents, with feelings of uncertainty, fear, anguish, ambivalence, anxiety, boredom, lack of motivation, depression, and situations of suicidal ideation standing out. Intensifying the use of the internet, especially the consumption of news related to the pandemic, caused discomfort in the adolescents and aggravated negative feelings. Family rela-tionships were fragmented and social support networks proved inconsistent, which became evident in the lack of embracement of the adolescents' psychosocial needs, who often resorted to social media looking for help. In addition, there were failures or deficiencies in the coping strategies formulated and adopted by the adolescents themselves to improve their well-being, which caused their mental distress to persist at a constant level and led to low productivity and motivation during the pandemic. It is expected that the findings of the present study stir reflections oriented toward actions that cover adolescent mental care during and after the COVID-19 pandemic and take into account the complexity of the social contexts in which this population is inserted.

## ASSOCIATE EDITOR

Thiago da Silva Domingos
